# Supplementary material for: Dissecting the properties of circulating IgG against streptococcal pathogens through a combined systems antigenomics-serology workflow
Source: Nat Commun. 2025 Feb 24;16:1942. doi: 10.1038/s41467-025-57170-5 (PMC11850916; doi:10.1038/s41467-025-57170-5)
Supplement: Supplementary file 1 — Supplementary Information [file 41467_2025_57170_MOESM1_ESM.pdf]

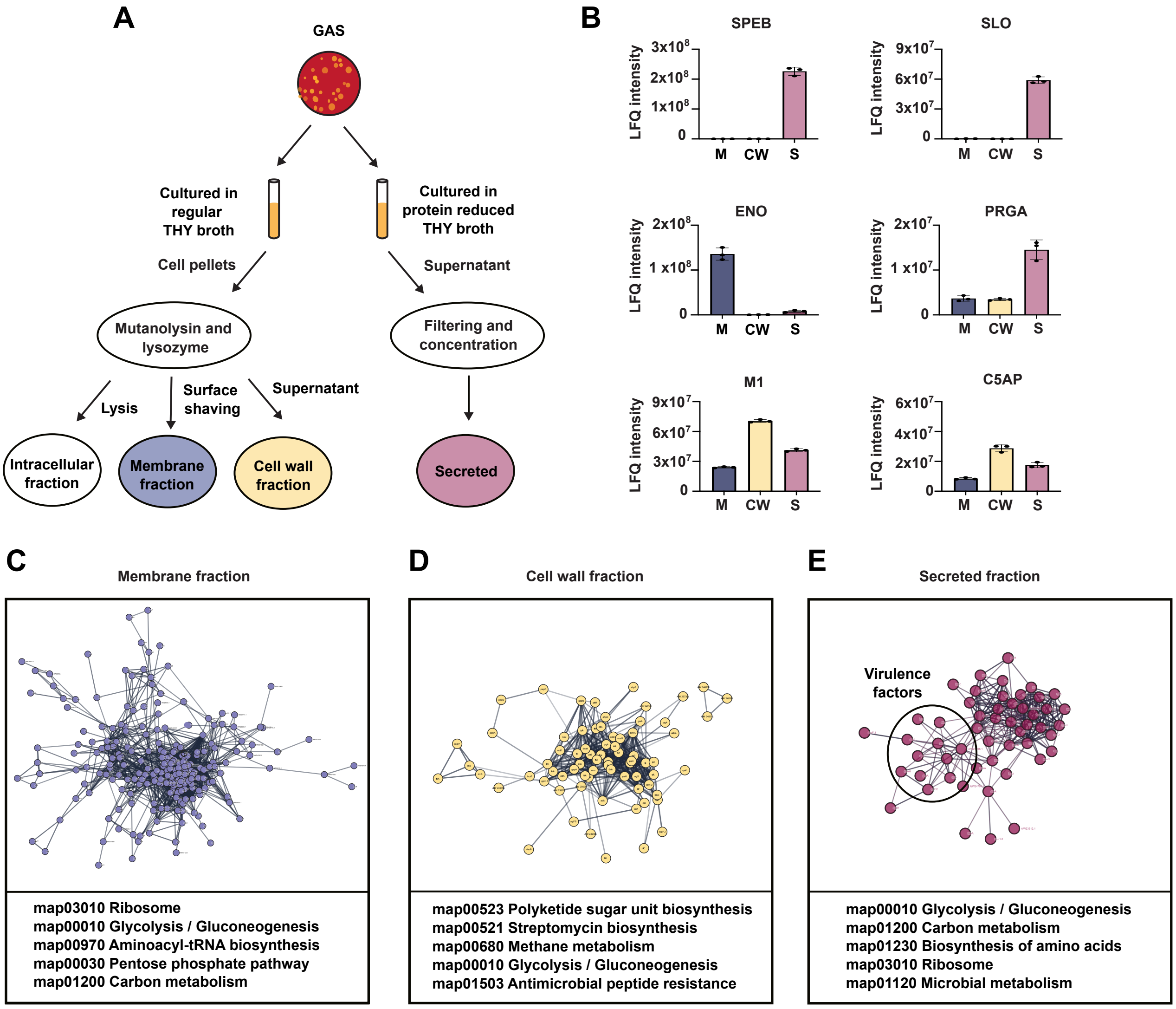

Fig. S1: Cellular localization and network analysis of the SF370 GAS proteome. (A) Schematic summary showing the bacterial growth conditions and different steps involved in the fractionation method to obtain secreted, cell wall and membrane fractions. **(B)** Bar plots for GAS proteins (SPEB, SLO, ENO, PRGA, M1 and C5AP) representing the difference in their abundances based on LFQ intensity across membrane (M), cell wall (CW) and secreted (S) fractions. Bars represent mean values and error bars represent standard deviation (SD). STRING functional analysis of protein networks of proteins identified in **(C)** membrane, **(D)** cell wall and **(E)** secreted fraction.

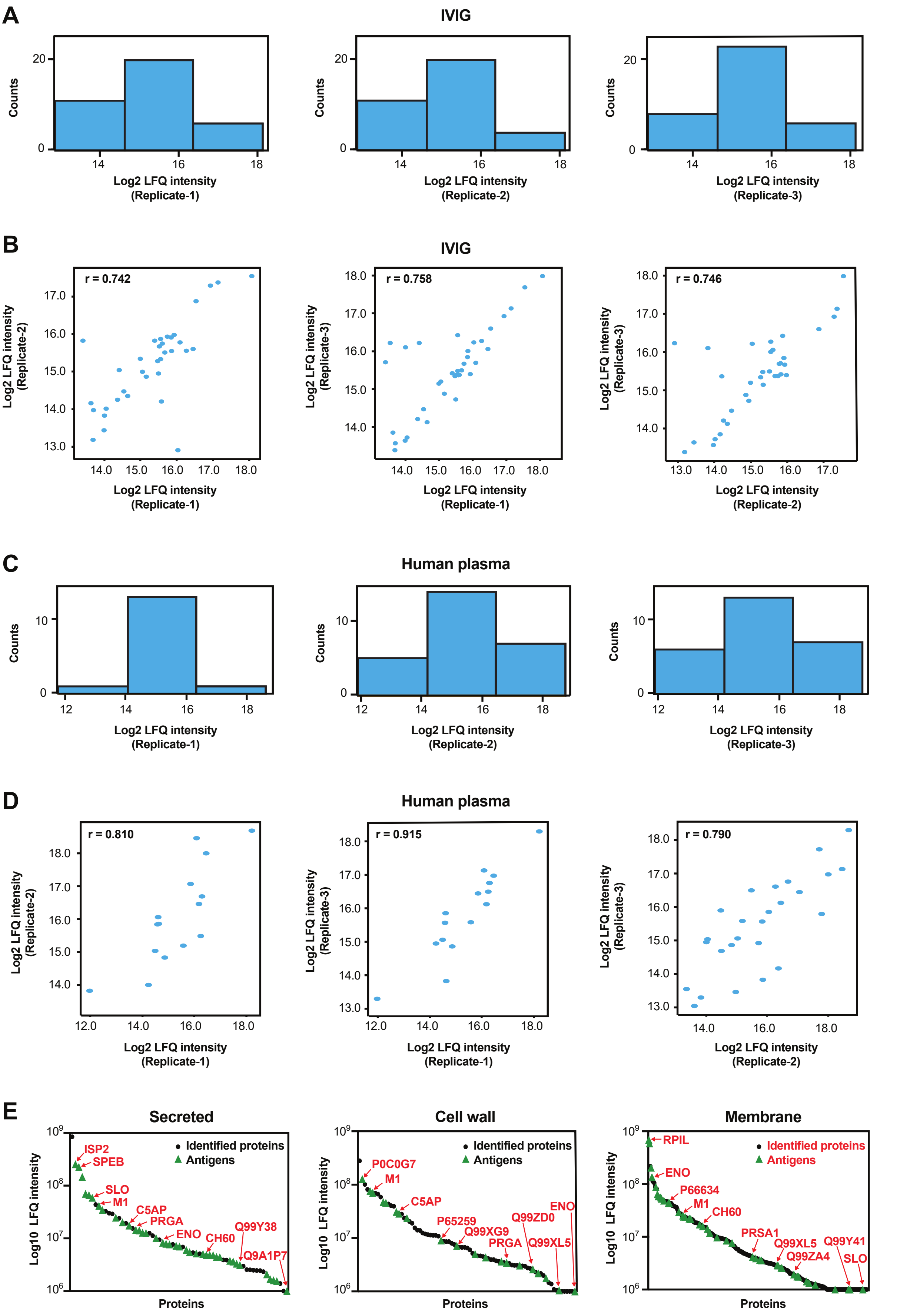

Fig. S2: Reproducibility and dynamic range of the antigen identification workflow. Distribution of antigens identified by IgG from **(A)** IVIG and **(C)** human plasma across three replicates. Pearson correlation plots of the intensity of the antigens demonstrating strong correlation between the replicates for **(B)** IVIG and **(D)** human plasma. **(E)** Waterfall plots depicting the abundance distribution of GAS proteins and identified antigens in secreted, cell wall and membrane fractions of SF370.

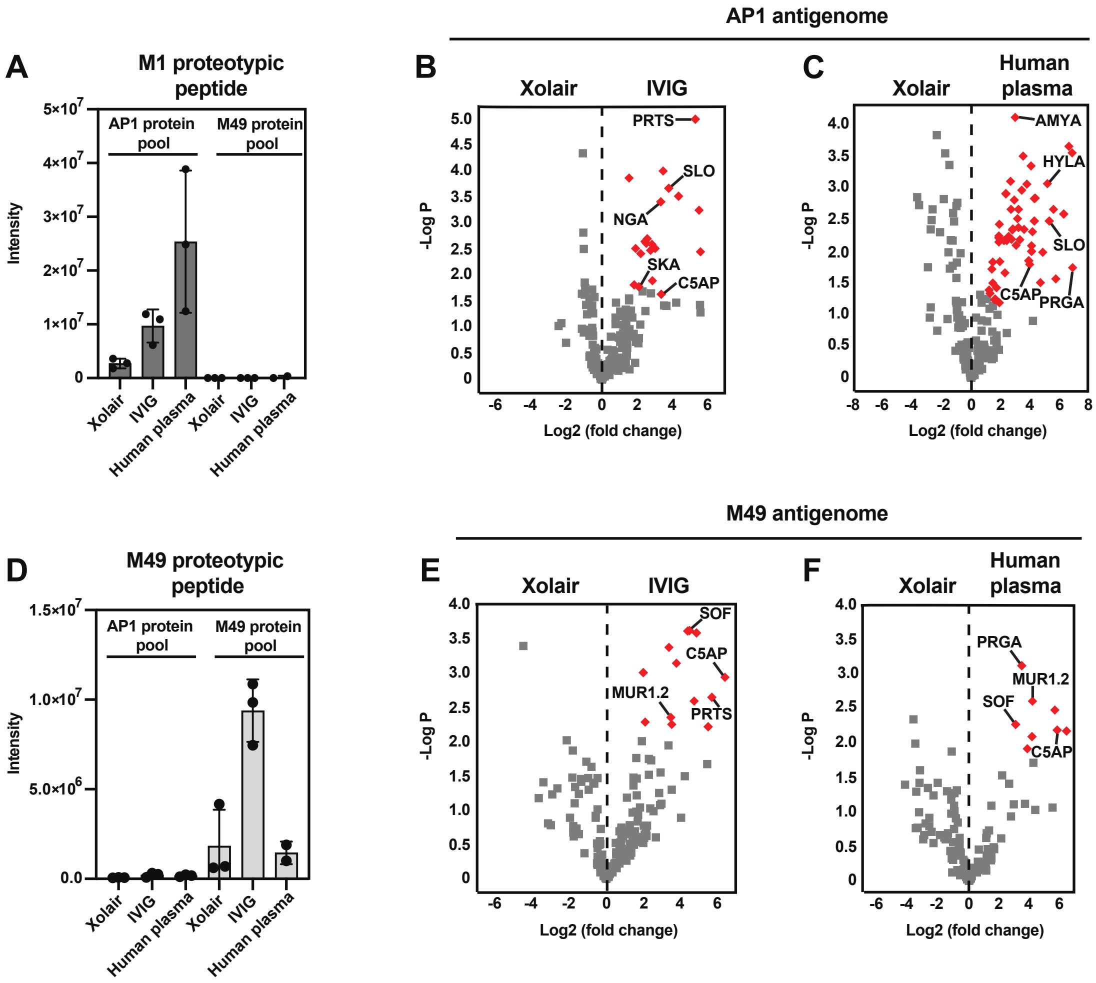

Fig. S3: **AP1 and M49 specific IgG antigenome. (A)** M1 proteotypic peptide enrichment from AP1 and M49 protein pools using Xolair, IVIG and pooled human plasma. Volcano plot displaying significant antigens recognized by **(B)** IVIG and **(C)** pooled human plasma from AP1 protein fraction. (**D)** M49 proteotypic peptide enrichment from AP1 and M49 protein pool using Xolair, IVIG and pooled human plasma. Volcano plot displaying significant antigens recognized by **(E)** IVIG and **(F)** pooled human plasma from M49 protein fraction. The data summarize the results from 2-3 technical replicates. Statistical significance was determined using a both side t-test with an FDR of 0.05 to correct for multiple comparisons. Bars represent mean values and error bars represent SD.

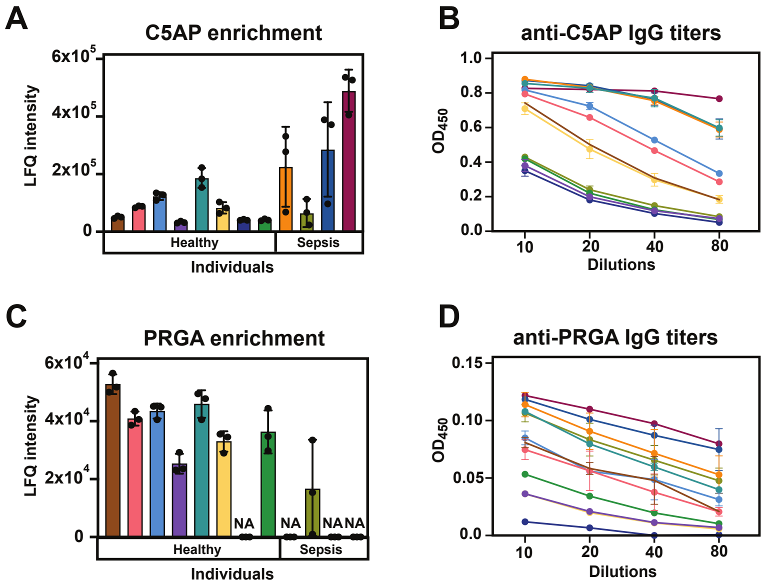

Fig. S4: GAS antigen enrichment and titers across different individuals. (A) C5AP and **(C)** PRGA enrichment across healthy and sepsis individuals. **(B)** anti-C5AP and **(D)** anti-PRGA IgG titers for healthy and sepsis individuals. Bars represent mean values and error bars represent SD.

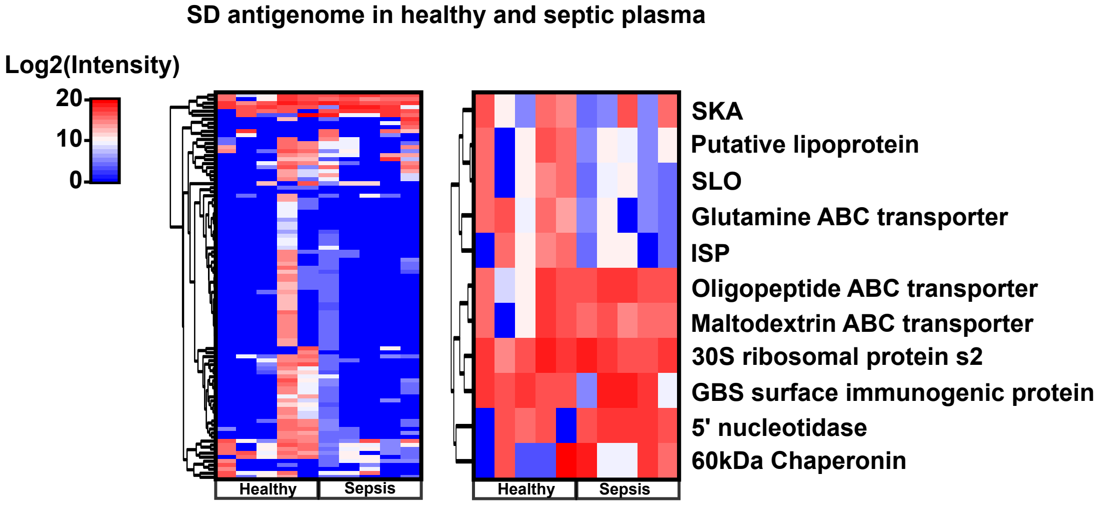

Fig. S5: Differential expression of log2 intensity of SD antigenome across plasma from healthy individuals and convalescent plasma from septic individuals infected with stG62647 isolate of SD.

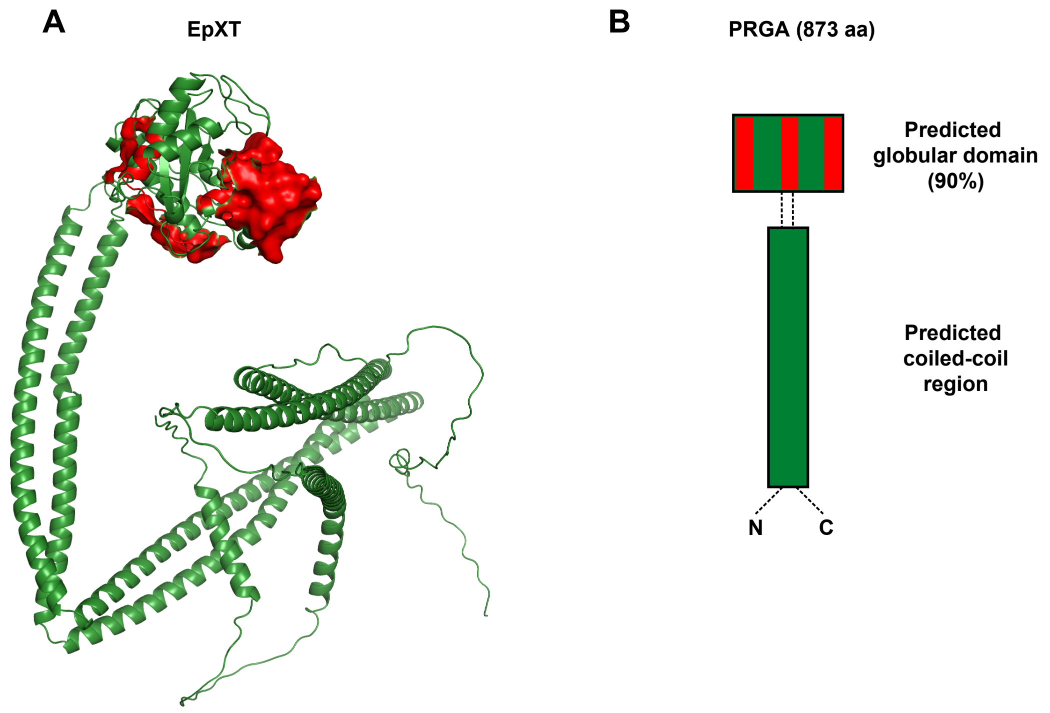

Fig. S6: Epitope mapping of PRGA. (A) Identified epitopes (marked red) by EpXT displayed on the PRGA model. **(B)** Relative peptide intensity (%) of the identified epitopes mapped onto a PRGA cartoon.

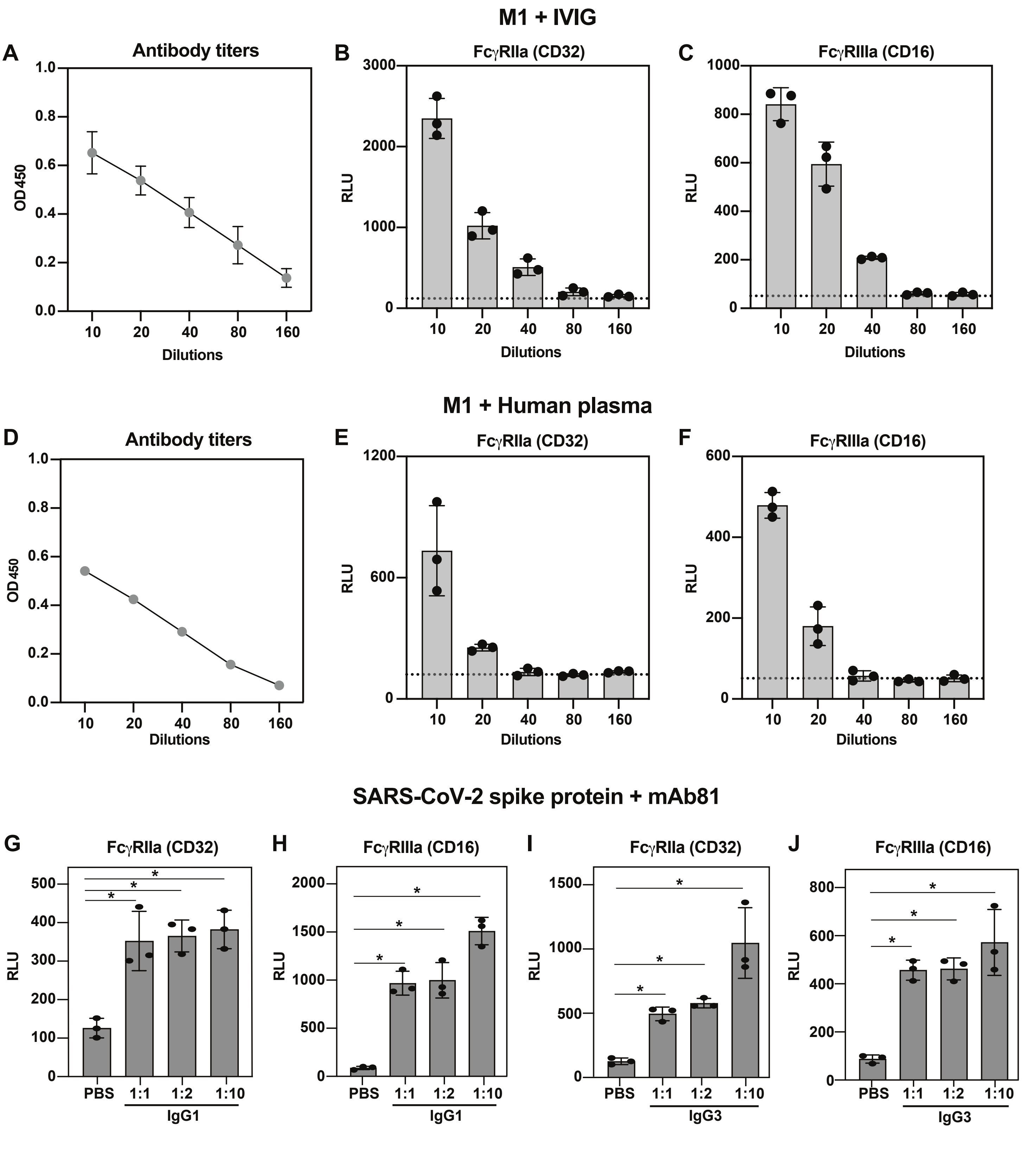

Fig. S7: Dose-dependent activation of FcγR by anti-M1 antibodies circulating in IVIG and pooled human plasma and by SARS-Cov-2 anti-spike monoclonal antibody 81 (mAb81). **(A)** anti-M1 titers in IVIG. **(B)** FcγRIIa (CD32) and **(C)** FcγRIIIa (CD16) activity assay of M1 with different dilutions of IVIG. Dotted lines indicate the background values. **(D)** anti-M1 titers in pooled human plasma. **(E)** FcγRIIa (CD32) and **(F)** FcγRIIIa (CD16) activity assay of M1 with different dilutions of pooled human plasma. Dotted lines indicate the background values. **(G)** FcγRIIa (CD32) and **(H)** FcγRIIIa (CD16) activity assay of SARS-CoV-2 spike protein with anti-spike mAb81 in IgG1 scaffold. **(I)** FcγRIIa (CD32) and **(J)** FcγRIIIa (CD16) activity assay of SARS-CoV-2 spike protein with anti-spike mAb81 in IgG3 scaffold. Statistical significance was assessed by ordinary one-way ANOVA with Dunnett's multiple comparisons test, * $p<0.05$. Bars represent mean values and error bars represent SD.

SF370 + mAb25

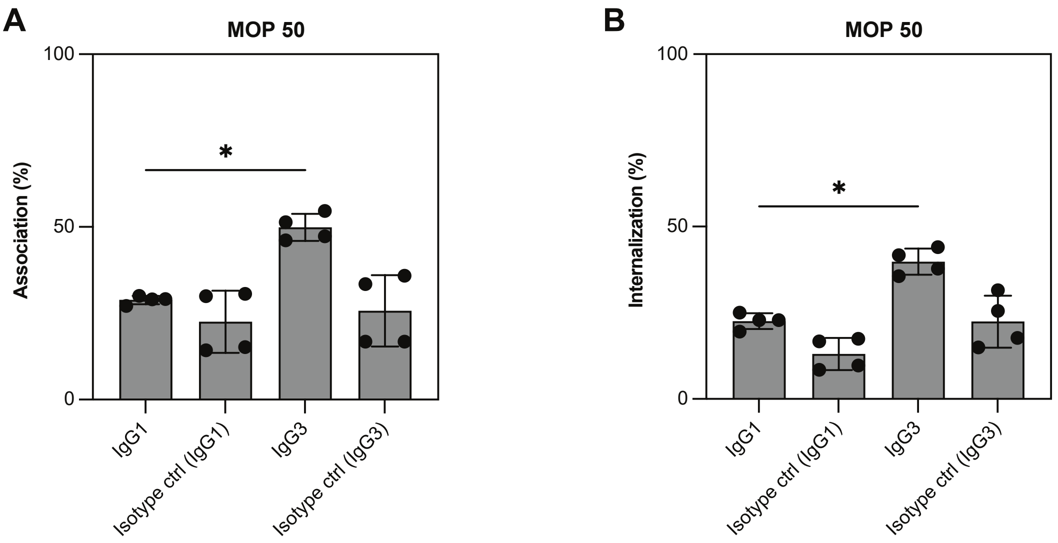

Fig. S 8: THP -1 cell mediated phagocytosis of SF370 with mAb25. Bar graphs represent percentage of THP-1 cells **(A)** associated and **(B)** internalized with SF370 for MOP -50. Four technical replicates were used, and statistical significance was assessed by unpaired t test with Welch's correction, * $p<0.05$. Bars represent mean values and error bars represent SD.
