# Supplementary material for: Dissecting the properties of circulating IgG against streptococcal pathogens through a combined systems antigenomics-serology workflow
Source: Nat Commun. 2025 Feb 24;16:1942. doi: 10.1038/s41467-025-57170-5 (PMC11850916; doi:10.1038/s41467-025-57170-5)
Supplement: Supplementary file 3 — Description of Additional Supplementary Files [file 41467_2025_57170_MOESM3_ESM.pdf]

Description of Additional Supplementary files

Supplementary data 1: Proteins identified in secreted, cell wall, membrane and intracellular GAS and SD fractions.

Supplementary data 2: Average LFQ intensities (across triplicates) of significantly enriched antigens from SF370, AP1 and M49 lysates using IgG from IVIG and pooled human plasma.

Supplementary data 3: Average LFQ intensities (across triplicates) of GAS antigens from SF370 and identified using plasma from healthy and sepsis individuals.

Supplementary data 4: LFQ intensities (across triplicates) of unique and common antigens enriched with IVIG and HP using GAS and SD lysates, with their homologous proteins and the homology percentage.

Supplementary data 5: Average log2 LFQ intensities (across triplicates) of SD antigens identified from stG62647 lysates using plasma from healthy and convalescent plasma from septic individuals.

Supplementary data 6: Identified epitopes, their length, start position, end position and corresponding intensity for C5AP, PRGA and M1.

Supplementary data 7: Experimental and uptake details for all observed peptides in HDX-MS analysis.

Supplementary data 8. HDX-MS standard report table.

Supplementary data 9: Glycoproteomics analysis of antigen specific IgG in IVIG.
