# Supplementary material for: Dissecting the properties of circulating IgG against streptococcal pathogens through a combined systems antigenomics-serology workflow
Source: Nat Commun. 2025 Feb 24;16:1942. doi: 10.1038/s41467-025-57170-5 (PMC11850916; doi:10.1038/s41467-025-57170-5)
Supplement: Supplementary file 12 — Supplementary data 9 [file 41467_2025_57170_MOESM12_ESM.pdf]

Dataset 8. IgG glycoforms modifying antigen-specific antibodies in IVIG and expressed as percentage of total glycopeptide intensity.

Glycoforms	IVIG_IgG1	IVIG_IgG2	IVIG_IgG3/4	M1_IgG1	M1_IgG2	M1_IgG3/4	C5AP_IgG1	C5AP_IgG2	PRGA_IgG1	PRGA_IgG2
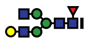	34.8	37.2	34.9	44.2	38	32.4	43.5	34.2	43.1	33.9
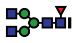	38	44	54.3	29.9	48.6	57.8	30.6	53.6	38.6	54.1
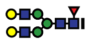	12.1	12.5	8.5	14.2	9.8	8.5	12.8	9.1	10.3	8.2
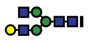	3.6	0	0	4.9	0	0	3.1	0	2.7	0
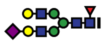	5.2	2.5	0.9	3.8	1.9	1.3	5	2	2.4	2.4
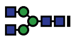	1.8	1.3	0	2.2	0	0	3.9	0	2.6	0
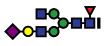	1.2	2.5	1.4	0.6	1.7	0	0.9	1.2	0.3	1.5
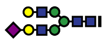	0.4	0.1	0	0.2	0	0	0.1	0	0	0
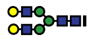	2.7	0	0	0	0	0	0	0	0	0
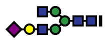	0.1	0.1	0	0	0	0	0	0	0	0

◆ N-acetylneuraminic acid ○ Galactose ● Mannose

■ N-acetylglucosamine ▲ Fucose
