# Supplementary figures and images for: Dissecting the properties of circulating IgG against streptococcal pathogens through a combined systems antigenomics-serology workflow
Source: Nat Commun. 2025 Feb 24;16:1942. doi: 10.1038/s41467-025-57170-5 (PMC11850916; doi:10.1038/s41467-025-57170-5)

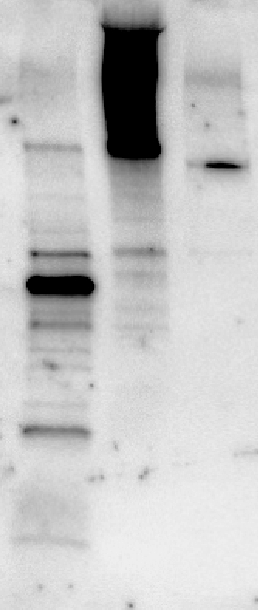

Supplement: Supplementary file 14 — Source data [file 41467_2025_57170_MOESM14_ESM.zip › WB_Fig 1E left.tif]

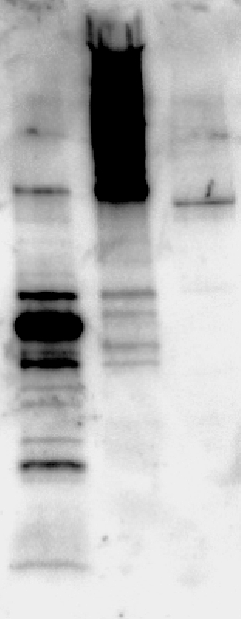

Supplement: Supplementary file 14 — Source data [file 41467_2025_57170_MOESM14_ESM.zip › WB_Fig 1E right.tif]
